# Insights into [(2-phen­oxy­phen­yl)lithium(THF)(TMEDA)] and diphenyl ether: structural influence of metalation on the aromatic system

**DOI:** 10.1107/S2056989026002756

**Published:** 2026-05-07

**Authors:** Michael Nuss, Tobias Schrimpf, Carsten Strohmann

**Affiliations:** a Technische Universität Dortmund, Fakultät Chemie und Chemische Biologie, Otto-Hahn-Strasse 6, 44227 Dortmund, Germany; Illinois State University, USA

**Keywords:** crystal structure, Hirshfeld surface analysis, metalation, li­thia­tion, lithium chemistry

## Abstract

The title compound, (2-phen­oxy­phenyl-κ*C*^1^)(tetra­hydro­furan-κ*O*)(*N*,*N*,*N*′,*N*′-tetra­methyl­ethylenedi­amine-κ^2^*N*,*N*′)lithium, [Li(C_12_H_9_O)(C_6_H_16_N_2_)(C_4_H_8_O)] or [(2-phen­oxy­phen­yl)lithium(THF)(TMEDA)], (**1**), is a monomeric lithium complex in which tetra­hydro­furan (THF) and *N*,*N*,*N*′,*N*′-tetra­methyl­ethylenedi­amine (TMEDA) play essential roles in stabilizing the lithium ion and promoting a monomeric aggregate. This stabilization allows the lithium cation to maintain a distorted tetra­hedral coordination environment, which enhances its reactivity in organometallic chemistry. Organolithium species, particularly in combination with TMEDA and THF, are widely used in synthetic organic chemistry for deprotonation and metalation reactions due to their strong nucleophilic and basic properties. The structure of compound **1**, [Li(C_12_H_9_O)(C_6_H_16_N_2_)(C_4_H_8_O)]·0.212[C_5_H_12_]·0.2[C_5_H_12_], was determined at 100 K and crystallizes in the triclinic space group *P*1. The structure exhibits disorder of the coordinating THF ligand as well as disordered solven, which was modeled using a split model. In addition, disordered co-crystallized *n*-pentane was treated using the *OLEX2* solvent-mask procedure. For comparison, diphenyl ether (**2**), C_12_H_10_O, was also redetermined at 100 K and refined using *SHELXL*. It crystallizes in the ortho­rhom­bic space group *P*2_1_2_1_2_1_. This redetermination enables a direct and consistent comparison between educt and product and highlights the influence of li­thia­tion on structural properties. The structure of diphenyl ether corresponds to the literature-known polymorph II reported by Choudhury *et al.* [(2004). *J. Am. Chem. Soc.***126**, 12274–12275; CSD code: RAFFIO], and serves as a reliable reference for structural comparison.

## Abstract 2

The title compound, (2-phen­oxy­phenyl-κ*C*^1^)(tetra­hydro­furan-κ*O*)(*N*,*N*,*N*′,*N*′-tetra­methyl­ethylenedi­amine-κ^2^*N*,*N*′)lithium, [Li(C_12_H_9_O)(C_6_H_16_N_2_)(C_4_H_8_O)] or [(2-phen­oxy­phen­yl)lithium(THF)(TMEDA)] is a monomeric lithium complex in which tetra­hydro­furan (THF) and *N*,*N*,*N*′,*N*′-tetra­methyl­ethylenedi­amine (TMEDA) play essential roles in stabilizing the lithium ion and promoting a monomeric aggregate. For comparison, diphenyl ether was also redetermined at 100 K.

## 1. Chemical context

Organolithium compounds are indispensable reagents in organic synthesis and are highly valued for their strong nucleophilic and basic properties. These versatile reagents play a crucial role in various transformations, including metalation and nucleophilic addition reactions, making them essential for constructing complex organic mol­ecules. However, a significant challenge in the use of organolithium reagents is their strong tendency to form aggregates in solution, which can decrease their reactivity and limit selectivity (Gessner *et al.*, 2009 ▸). To counter this, the use of stabilizing ligands such as THF (tetra­hydro­furan) (Kleinheider *et al.*, 2024 ▸) and TMEDA (tetra­methyl­ethylenedi­amine) (Schrimpf *et al.*, 2022 ▸) has become widespread in organolithium chemistry. These ligands effectively break up aggregates and stabilize lithium ions in monomeric or other lower aggregates, enhancing both reactivity and selectivity. This stabilization is particularly useful when working with weakly coordinating or sterically hindered anions, such as the phen­oxy­phenyl group in the title compound. Using *t*-butyl­lithium instead of *n*-butyl­lithium enabled selective monoli­thia­tion of diphenyl ether, contrasting the known double-li­thia­tion observed under other conditions (Ogle *et al.*, 1997 ▸). The reaction of diphenyl ether (**2**) and *tert*-butyl lithium to the product **1** is shown in the scheme below.
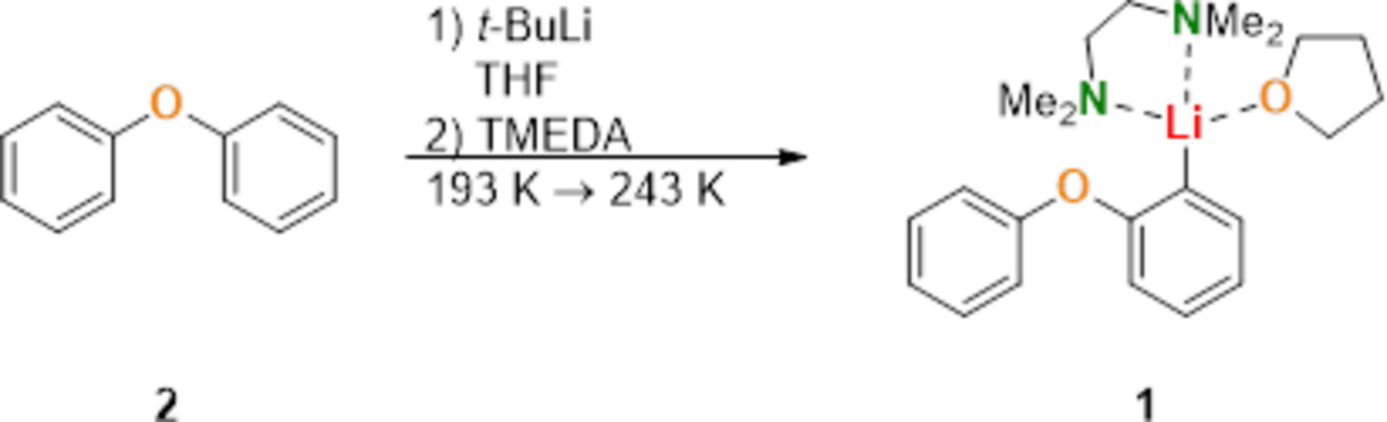


The resulting compound [(2-phen­oxy­phen­yl)lithium(THF)(TMEDA)] (**1**) exemplifies a monomeric organolithium species in which both ligands contribute to a distorted tetra­hedral geometry around lithium. This behavior aligns with directed *ortho*-metalation (*DoM*) strategies (Ebden *et al.*, 1995 ▸; Fleming *et al.*, 2011 ▸) and the complex-induced proximity effect (*CIPE*), which facilitate regioselective li­thia­tion by preorganizing the reactive sites (Whisler *et al.*, 2004 ▸). The proposed reaction mechanism for the DoM-li­thia­tion of diphenyl ether is shown in the scheme below.
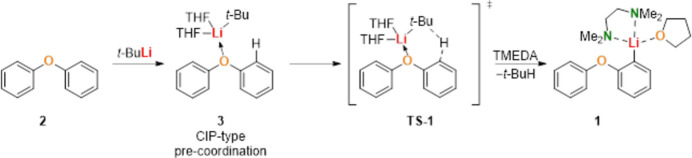


## 2. Structural commentary

**Figure 1:**
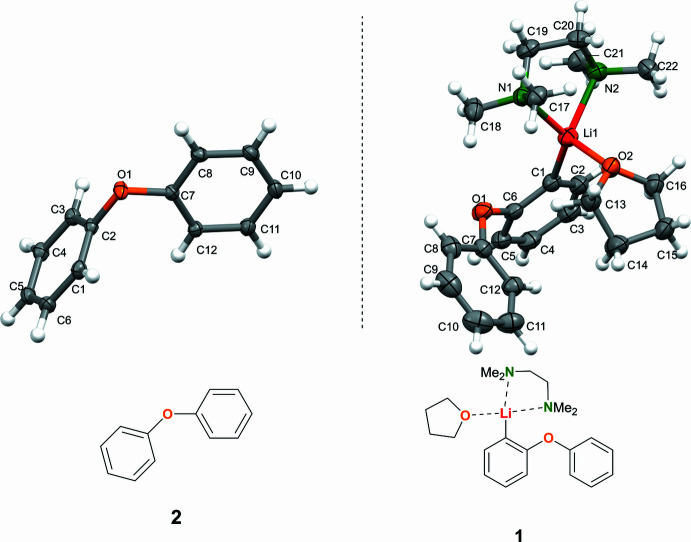
Mol­ecular structures of compound **1** ([(2-phenoxyphenyl)lithium(THF)(TMEDA)]) and diphenyl ether (**2**) shown separately. Displacement ellipsoids are drawn at the 50% probability level. Hydrogen atoms are omitted for clarity.

The crystal structures of diphenyl ether (**2**) and its mono-li­thia­ted derivative, [(2-phen­oxy­phen­yl)lithium(THF)(TMEDA)] (**1**) (Fig. 1 ▸), were determined by single-crystal X-ray diffraction. Compound **1** was measured using Cu *K*α radiation, while compound **2** was measured using Mo *K*α radiation.

The coordinating THF ligand in **1** is disordered and was modeled using a split model with occupancies constrained to sum to unity. The major and minor components have refined occupancies of 0.66 and 0.34, respectively. The disordered solvent was treated with the *OLEX2* solvent mask procedure, which revealed two solvent-accessible voids of 421 Å^3^ per unit cell, containing 71 electrons. This corresponds to approximately 0.4 mol­ecules of pentane per asymmetric unit.

Comparative analysis of selected bond lengths and angles indicates subtle structural changes upon lithiation. In particular, the C—O bond associated with the phenoxy unit is slightly increased in compound **1** compared to diphenyl ether** (2**), consistent with coordination to the lithium center. Selected geometric parameters are compiled in Tables 1 ▸–3 ▸ ▸.

The dihedral angles between the phenyl rings are 88.43 (7)° in diphenyl ether (**2**), consistent with polymorphs II (87.6°) and I [88.4 (7)°], and 75.42 (8)° in compound **1**. This indicates that the rings remain close toperpendicular in both structures, with a slight decrease in the dihedral angle upon li­thia­tion (Choudhury *et al.*, 2004 ▸). This distortion underscores the electronic and steric influence of the lithium coordination.

## 3. Supra­molecular features

**Figure 2:**
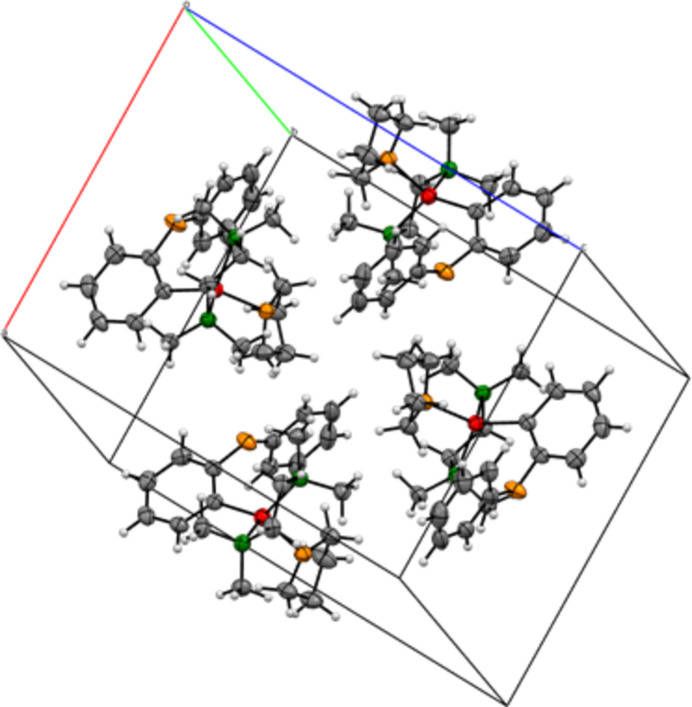
Packing of compound **2** with displacement ellipsoids drawn at the 50% probability level. Disorder at the thf mol­ecule was omitted for clarity.

**Figure 3:**
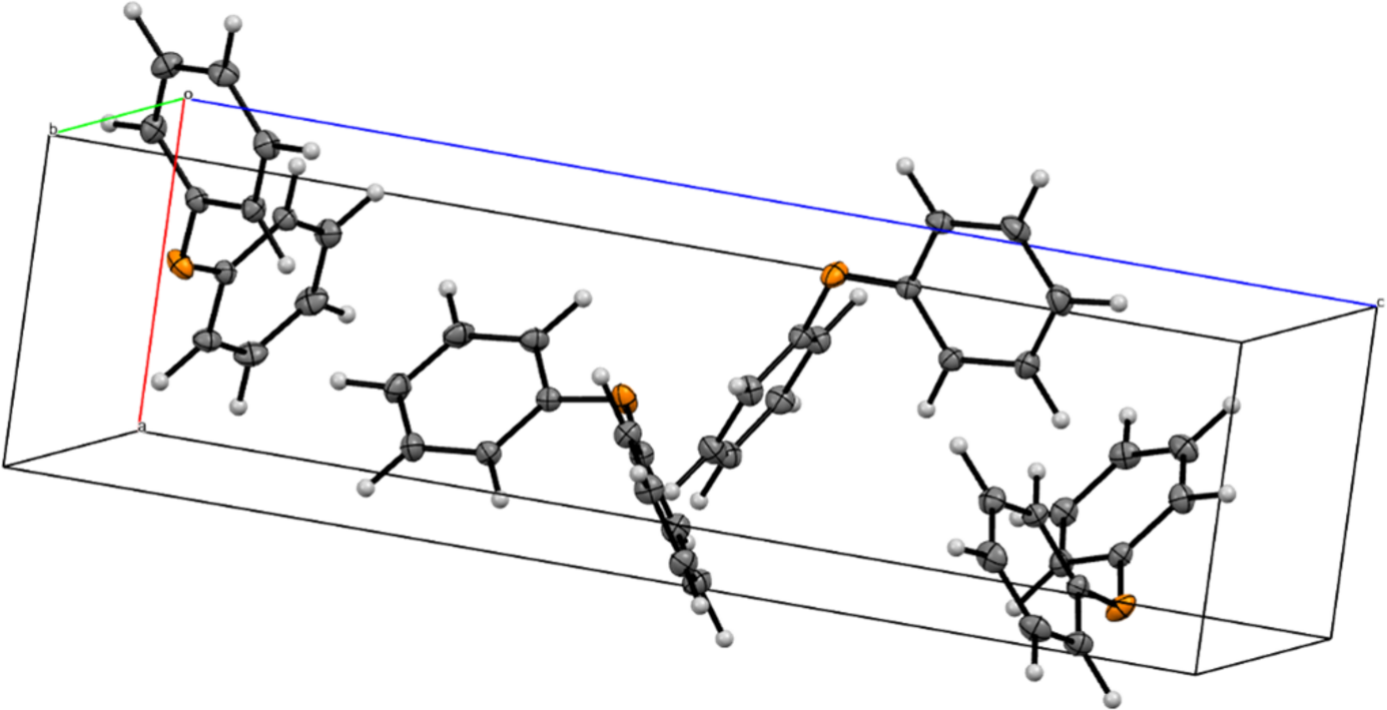
Packing of compound **1**.

The crystal structure of diphenyl ether (**2**) (Fig. 2 ▸) is consistent with the literature-reported polymorph II (Choudhury *et al.*, 2004 ▸), displaying comparable inter­molecular inter­actions and packing motifs. This provides a meaningful structural reference for assessing the impact of li­thia­tion in compound **1**. The packing of **1** is shown in Fig. 3 ▸

**Figure 4:**
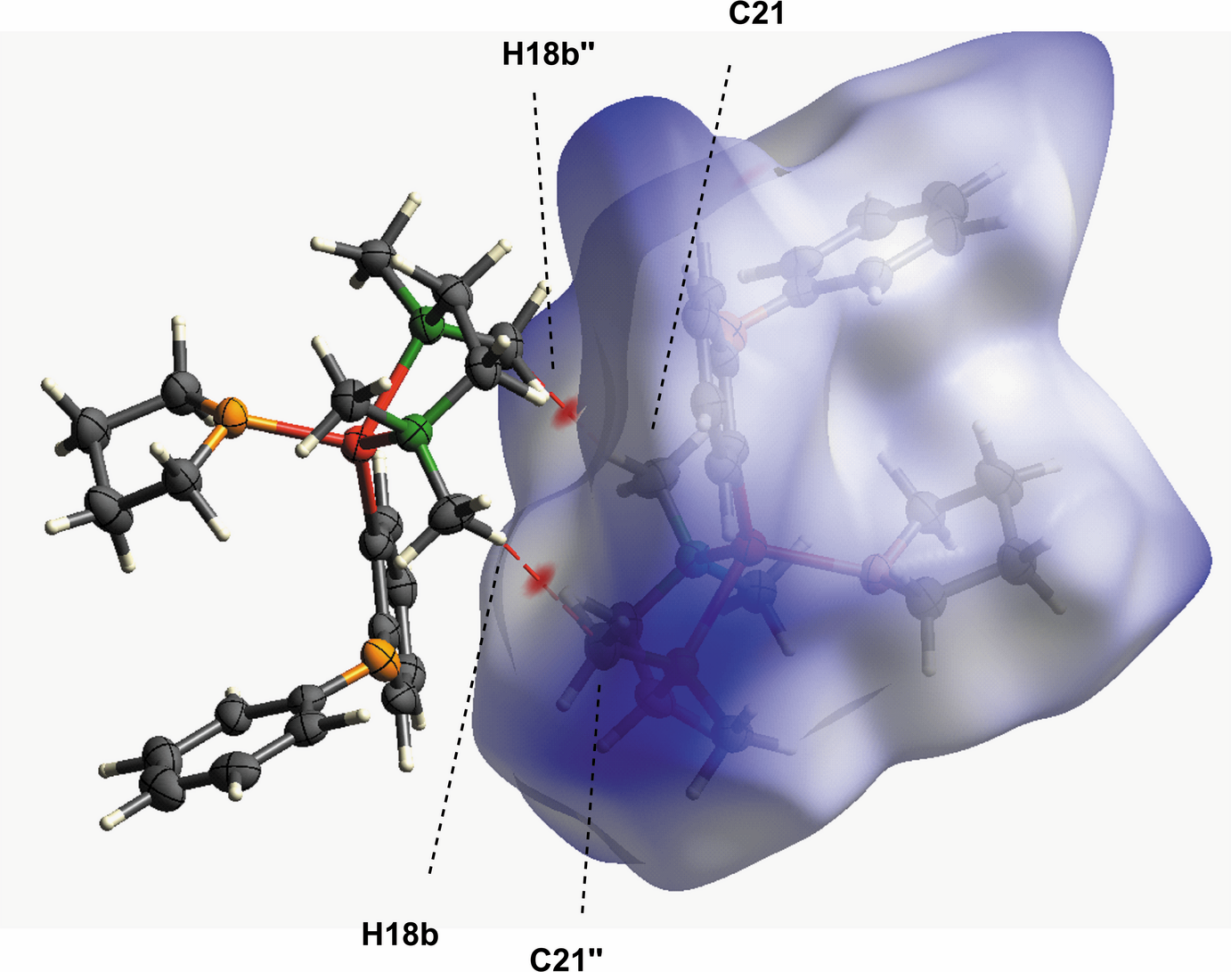
Hirshfeld surface of compound **1** mapped over *d*_norm_, highlighting dominant H⋯C intermolecular contacts.

The crystal structure of [(2-phen­oxy­phen­yl)lithium(THF)(TMEDA)] (**1**) reveals that the mol­ecular packing is largely governed by weak van der Waals inter­actions. A detailed Hirshfeld surface analysis, (Spackman & Jayatilaka, 2009 ▸) was performed on the major disorder component in the asymmetric unit, highlighting the significance of these weak inter­actions. Figs. 4 ▸ and 5 ▸ illustrate the Hirshfeld surface mapped over *d*_norm_ and the related fingerprint plots generated b*y CrystalExplorer* (Spackman *et al.*, 2021 ▸; McKinnon *et al.*, 2007 ▸) are shown in Fig. 6 ▸.

**Figure 5:**
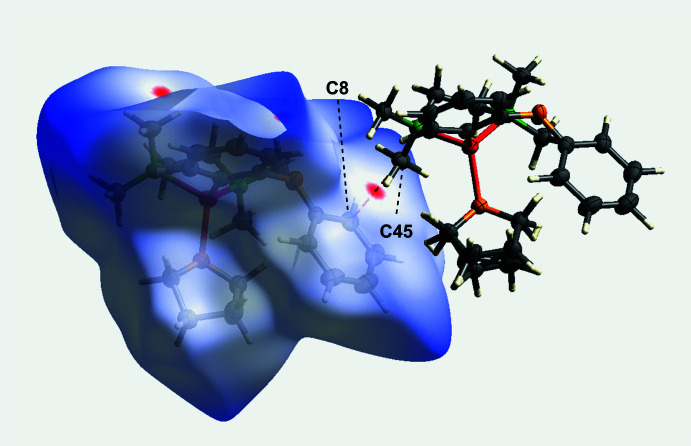
Hirshfeld surface of compound **1** mapped over *d*_norm_, highlighting C—C-based intermolecular contacts.

**Figure 6:**
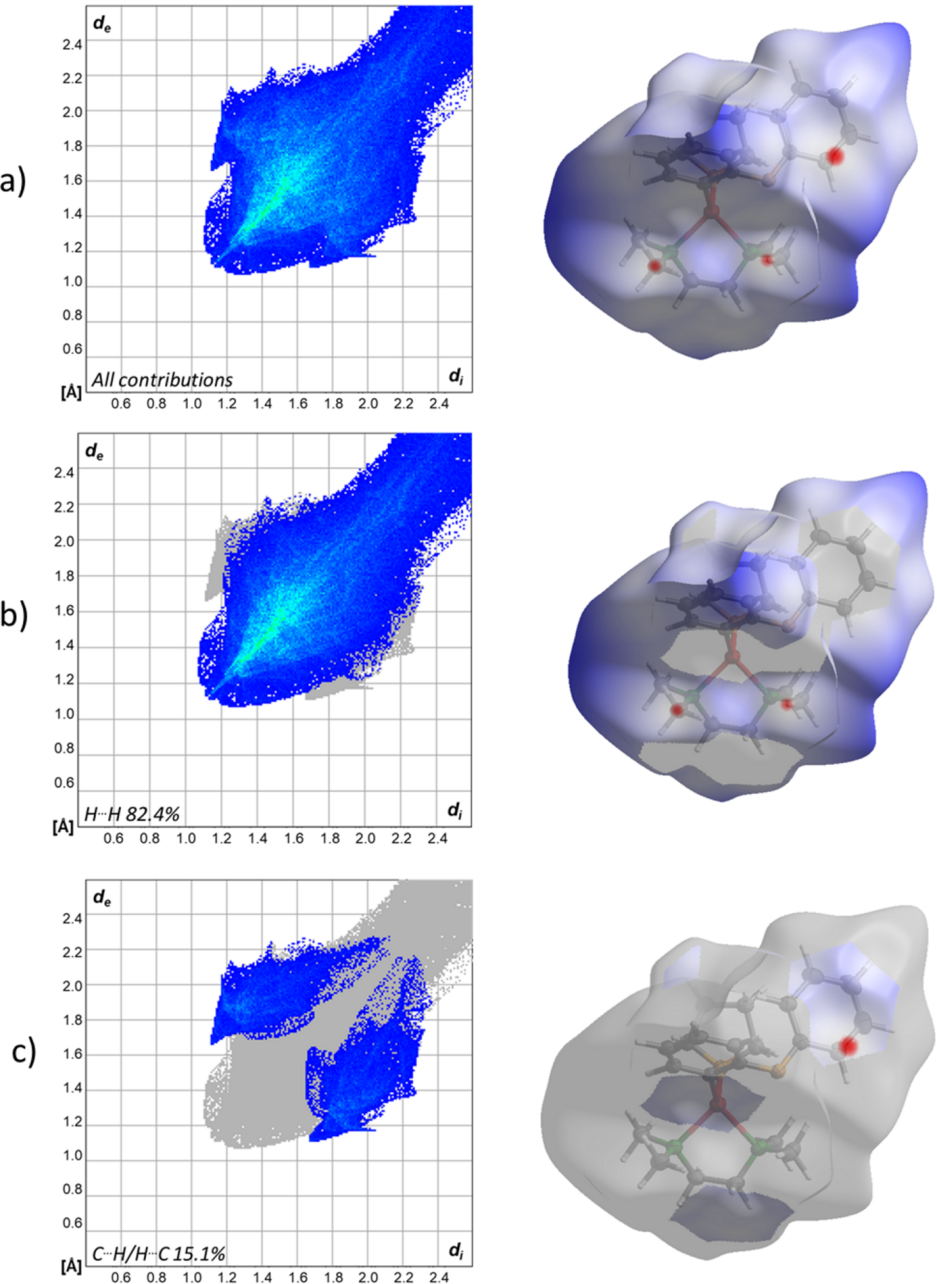
Two-dimensional fingerprint plots of [(2-phen­oxy­phen­yl)lithium(THF)(TMEDA)], (*a*) showing all contributions, (*b*) showing the H⋯H contributions and (*c*) showing the contributions of carbon and hydrogen (blue areas). The corresponding surfaces obtained by Hirshfeld surface analysis are also displayed.

H⋯H van der Waals contacts dominate the crystal structure, constituting 82.4% of the close inter­actions and significantly contributing to the overall packing. In addition, short intermolecular contacts involving the TMEDA ligand further support the crystal packing (see Figs. 4 ▸ and 5 ▸). In particular, the short intermolecular H⋯C van der Waals contact H18*B*⋯C21(2 − *x*, 2 − *y*, 1 − *z*) of 2.683 (6) Å, which is 0.22 Å shorter than the sum of the van der Waals radii, provides a representative example of such close contacts. Additional short intermolecular contacts are also present. Figure 5 ▸ illustrates a close C⋯C van der Waals contact C45⋯C8(*x*, *y* + 1, *z* − 1) of 3.305 (3) Å, 0.10 Å shorter than the sum of the van der Waals radii.

The inter­actions between TMEDA’s methyl groups and the aromatic system of the diphenyl ether further contribute to the packing stability, even in the absence of strong hydrogen bonding or π-stacking.

The Hirshfeld surface, with rescaled surface properties ranging from −0.055 to 3.298 arbitrary units, underscores the importance of these weak inter­actions. The combination of H⋯H and C—H⋯O inter­actions, though individually weak, results in a cohesive packing arrangement that consolidates the crystal.

## 4. Database survey

A search of the Cambridge Structural Database (WebCSD, October 2024; Groom *et al.*, 2016 ▸) was conducted to identify structures with fragments similar to those found in [(2-phen­oxy­phen­yl)lithium(THF)(TMEDA)].

When focusing on carbanion structures coordinated by both TMEDA and THF ligands, three closely related structures were identified: benzyl-(*N*,*N*,*N*′,*N*′-tetra­methyl­ethylenedi­amine-*N*,*N*′)tetra­hydro­furan­lithium (CSD refcode VEGWEJ; Zarges *et al.*, 1989 ▸), (2-phenyl-1,3-di­thian-2-yl)(tetra­hydro­furan)(*N*,*N*,*N*′,*N*′-tetra­methyl­ethane-1,2-di­amine)­lithium (LIP­THF; Amstutz *et al.*, 1981 ▸), and [1-chloro-2,2-bis­(4-chloro­phen­yl)ethen­yl](*N*,*N*,*N*′,*N*′-tetra­methyl­ethylenedi­amine)](tetra­hydro­furan)­lithium tetra­hydro­furan solvate (PEMNOK; Boche *et al.*, 1993 ▸). All three feature a lithium cation coordinated by a carbanion, along with one mol­ecule of THF and one TMEDA mol­ecule. These structures are comparable to the title compound in terms of coordination geometry and ligand environment, providing good examples of how TMEDA and THF stabilize lithium–carbanion complexes in a similar coordination profile.

Inter­estingly, no structures featuring a phenyl­anion coordinated by both TMEDA and THF were found in the database, indicating the novelty of the title compounds combination of ligands and anion.

Additionally, several structures were identified that feature lithium complexes coordinated by THF without TMEDA but with three THF ligands, providing an inter­esting comparison for the role of TMEDA in these systems. These examples include *t*-butyl­tris­(tetra­hydro­furan)­lithium (POJVET; Kleinheider *et al.*, 2024 ▸), [α-(thiophenyl)benzyl-*C*]tris(tetrahydrofuran)lithium (JILRAX; Zarges *et al.*, 1991 ▸), (μ_2_-meth­yl)-bis­(η^5^-penta­methyl­cyclo­penta­dien­yl)methyl­tris­(tetra­hydro­furan)­lithiumlutetium (PAJKES; Thomson *et al.*, 2011 ▸) and 2,3,4,5-tetra­fluoro­phenyl­tris­(tetra­hydro­furan)­lithium (ZEL­DUP; Kottke *et al.*, 1995 ▸). These structures demonstrate how lithium cations can be stabilized by multiple THF mol­ecules in the absence of TMEDA, though the coordination geometry tends to vary slightly compared to complexes where TMEDA is present.

## 5. Synthesis and crystallization

Due to the air-sensitive nature of organolithium compounds, it was essential to carry out the procedure under an argon atmosphere using Schlenk techniques. Pre-dried and distilled tetra­hydro­furan (2.00 ml) and *n*-pentane (2.00 ml) were added to an evacuated 25 ml Schlenk flask, followed by diphenyl ether (0.34 g, 2.00 mmol, 1.00 eq.). After cooling the reaction mixture to 193 K, *t*-butyl­lithium (1.9 *M* in *n*-pentane, 1.26 ml, 2.40 mmol, 1.20 eq.) was added, followed by TMEDA (*N*,*N*′,*N*′,*N′*-tetra­methyl­ethylenedi­amine, 0.25 g, 2.20 mmol, 1.10 eq.). The resulting beige suspension was warmed to 243 K over a period of 2 h and then stored at 193 K. After approximately 10 days, colorless block-shaped crystals of the target compound (**1**) were obtained.

Single crystals of diphenyl ether (**2**) suitable for X-ray diffraction were obtained directly from the product bottle at ambient conditions (301 K). The compound was isolated without the use of any solvent. To promote crystallization, a sample was placed on a microscope slide and subjected to controlled cooling to lower temperatures. This process facilitated the formation of well-defined crystals, which were subsequently mounted and measured under suitable conditions for single-crystal X-ray diffraction analysis.

## 6. Refinement

Crystal data, data collection and structure refinement details are summarized in Table 4 ▸.

For compound **1**, disordered solvent contributions were treated using the *OLEX2* solvent-mask procedure. The mask revealed two solvent-accessible voids with a combined volume of 421 Å^3^ containing 71 electrons per unit cell. These were not modeled explicitly but are consistent with approximately 0.4 pentane mol­ecules per asymmetric unit. The coordinating THF mol­ecule was modeled using a split model with free occupancy refinement. The final occupancies refined to 0.66 and 0.34.

To ensure comparability, structure **2** was also refined using *SHELXL* (Sheldrick, 2015*b* ▸). All hydrogen atoms in **1** were positioned geometrically and refined using a riding model. In **2**, all hydrogen atoms were freely refined.

## Supplementary Material

Crystal structure: contains datablock(s) 1, 2. DOI: 10.1107/S2056989026002756/ej2012sup1.cif

Structure factors: contains datablock(s) 1. DOI: 10.1107/S2056989026002756/ej20121sup2.hkl

Structure factors: contains datablock(s) 2. DOI: 10.1107/S2056989026002756/ej20122sup3.hkl

CCDC references: 2537304, 2537303

Additional supporting information:  crystallographic information; 3D view; checkCIF report

## Figures and Tables

**Table 1:** Selected bond lengths (Å) and angles (°) for compound **1** and diphenyl ether (**2**)

Diphenyl ether		Compound **1**	
O1—C7	1.3784 (16)	O1—C7	1.363 (2)
O1—C2	1.3965 (16)	O1—C6	1.4290 (19)
C1—C2	1.386 (2)	C1—C6	1.386 (2)
C7—C12	1.3917 (17)	C7—C12	1.392 (3)
C2—O1—C7	117.94 (10)	C6—O1—C7	118.30 (13)
C8—C7—O1	155.27 (11)	C8—C7—O1	116.07 (16)
C12—C7—C8	120.94 (12)	C12—C7—C8	119.37 (16)

**Table 2:** Selected bond lengths (Å) and angles (°) for compound (**1**)

Bond		Angle	
C1—Li1	2.128 (3)	N1—Li1—N2	85.9 (1)
O2—Li1	2.023 (3)	N1—Li1—O2	102.63 (12)
N1—Li1	2.166 (3)	N2—Li1—O2	109.22 (12)
N2—Li1	2.156 (3)	C1—Li1—N1	129.96 (15)
C6—O1	1.4329 (19)	C1—Li1—N2	113.69 (13)
C7—O2	1.363 (2)	C1—Li1—O2	111.97 (14)

**Table 3:** Selected C—C bond lengths (Å) for compound **1** and diphenyl ether (**2**)

Diphenyl ether		Compound **1**	
C1—C2	1.386 (2)	C1—C6	1.386 (2)
C2—C3	1.3870 (18)	C6—C5	1.389 (2)
C3—C4	1.393 (2)	C5—C4	1.387 (3)
C4—C5	1.391 (2)	C4—C3	1.385 (2)
C5—C6	1.393 (2)	C3—C2	1.390 (2)

**Table 4:** Experimental details

	**1**	**2**
Crystal data		
Chemical formula	[Li(C_12_H_9_O)(C_6_H_16_N_2_)(C_4_H_8_O)]·0.5C_5_H_12_	C_12_H_10_O
*M*_r_	400.51	170.20
Crystal system, space group	Triclinic, *P* 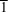	Orthorhombic, *P*2_1_2_1_2_1_
Temperature (K)	100	100
*a*, *b*, *c* (Å)	12.494 (4), 13.530 (4), 15.528 (5)	5.6154 (10), 7.7194 (11), 20.884 (3)
α, β, γ (°)	70.284 (11), 87.622 (10), 80.493 (10)	90, 90, 90
*V* (Å^3^)	2436.9 (14)	905.3 (2)
*Z*	4	4
Radiation type	Cu *K*α	Mo *K*α
μ (mm^−1^)	0.52	0.08
Crystal size (mm)	0.56 × 0.53 × 0.35	0.94 × 0.72 × 0.17

Data collection		
Diffractometer	Bruker SMART APEXII area detector	Bruker APEXII CCD
Absorption correction	Multi-scan (*SADABS*; Krause *et al.*, 2015 ▸)	Multi-scan (*SADABS*; Krause *et al.*, 2015 ▸)
*T*_min_, *T*_max_	0.444, 0.588	0.487, 0.566
No. of measured, independent and observed [*I* > 2σ(*I*)] reflections	78003, 9204, 7642	29791, 3439, 3113
*R*_int_	0.059	0.058
(sin θ/λ)_max_ (Å^−1^)	0.609	0.770

Refinement		
*R*[*F*^2^ > 2σ(*F*^2^)], *wR*(*F*^2^), *S*	0.053, 0.158, 1.08	0.039, 0.109, 1.06
No. of reflections	9204	3439
No. of parameters	513	158
H-atom treatment	H-atom parameters constrained	All H-atom parameters refined
Δρ_max_, Δρ_min_ (e Å^−3^)	0.48, −0.19	0.33, −0.18
Absolute structure	–	Flack *x* determined using 1169 quotients [(*I*^+^)−(*I*^−^)]/[(*I*^+^)+(*I*^−^)] (Parsons et al., 2013 ▸)
Absolute structure parameter	–	−0.8 (5)

Computer programs: *APEX2* and *SAINT* (Bruker, 2016 ▸), *SHELXT* (Sheldrick, 2015a), *SHELXT* (Sheldrick, 2015*a* ▸), *SHELXL2019/2* (Sheldrick, 2015*b* ▸) and *OLEX2* (Dolomanov *et al.*, 2009 ▸).

## supplementary crystallographic information

### (2-phenoxyphenyl-κC1)(tetrahydrofuran-κO)(N,N,N',N'-tetramethylethylenediamine-κ2N,N')lithium (1) Crystal data

**Table:**

[Li(C_12_H_9_O)(C_6_H_16_N_2_)(C_4_H_8_O)]·0.5C_5_H_12_	*Z* = 4
*M**_r_* = 400.51	*F*(000) = 876
Triclinic, *P*1	*D*_x_ = 1.092 Mg m^−^^3^
*a* = 12.494 (4) Å	Cu *K*α radiation, λ = 1.54178 Å
*b* = 13.530 (4) Å	Cell parameters from 3711 reflections
*c* = 15.528 (5) Å	θ = 13.1–70.1°
α = 70.284 (11)°	µ = 0.52 mm^−^^1^
β = 87.622 (10)°	*T* = 100 K
γ = 80.493 (10)°	Block, colourless
*V* = 2436.9 (14) Å^3^	0.56 × 0.53 × 0.35 mm

### (2-phenoxyphenyl-κC1)(tetrahydrofuran-κO)(N,N,N',N'-tetramethylethylenediamine-κ2N,N')lithium (1) Data collection

**Table:**

Bruker SMART APEXII area detector diffractometer	9204 independent reflections
Radiation source: microfocus sealed X-ray tube, Incoatec Iµs	7642 reflections with *I* > 2σ(*I*)
Mirror optics monochromator	*R*_int_ = 0.059
Detector resolution: 7.9 pixels mm^-1^	θ_max_ = 70.0°, θ_min_ = 3.0°
ω and φ scans	*h* = −15→15
Absorption correction: multi-scan (SADABS; Krause *et al.*, 2015)	*k* = −16→16
*T*_min_ = 0.444, *T*_max_ = 0.588	*l* = −18→18
78003 measured reflections	

### (2-phenoxyphenyl-κC1)(tetrahydrofuran-κO)(N,N,N',N'-tetramethylethylenediamine-κ2N,N')lithium (1) Refinement

**Table:**

Refinement on *F*^2^	Primary atom site location: dual
Least-squares matrix: full	Hydrogen site location: inferred from neighbouring sites
*R*[*F*^2^ > 2σ(*F*^2^)] = 0.053	H-atom parameters constrained
*wR*(*F*^2^) = 0.158	*w* = 1/[σ^2^(*F*_o_^2^) + (0.0916*P*)^2^ + 0.4885*P*] where *P* = (*F*_o_^2^ + 2*F*_c_^2^)/3
*S* = 1.08	(Δ/σ)_max_ = 0.001
9204 reflections	Δρ_max_ = 0.48 e Å^−^^3^
513 parameters	Δρ_min_ = −0.19 e Å^−^^3^
0 restraints	

### (2-phenoxyphenyl-κC1)(tetrahydrofuran-κO)(N,N,N',N'-tetramethylethylenediamine-κ2N,N')lithium (1) Special details

**Table:**

Geometry. All esds (except the esd in the dihedral angle between two l.s. planes) are estimated using the full covariance matrix. The cell esds are taken into account individually in the estimation of esds in distances, angles and torsion angles; correlations between esds in cell parameters are only used when they are defined by crystal symmetry. An approximate (isotropic) treatment of cell esds is used for estimating esds involving l.s. planes.

### (2-phenoxyphenyl-κC1)(tetrahydrofuran-κO)(N,N,N',N'-tetramethylethylenediamine-κ2N,N')lithium (1) Fractional atomic coordinates and isotropic or equivalent isotropic displacement parameters (Å^2^)

**Table:**

	*x*	*y*	*z*	*U*_iso_*/*U*_eq_	Occ. (<1)
O4	1.51935 (8)	1.31086 (8)	−0.24004 (7)	0.0346 (2)	
O2	0.94706 (9)	0.81436 (8)	0.26840 (7)	0.0358 (3)	
O1	1.13694 (9)	0.72823 (8)	0.52032 (8)	0.0397 (3)	
O3	1.60529 (9)	1.23372 (8)	0.04750 (9)	0.0411 (3)	
N3	1.62041 (10)	1.47301 (9)	−0.15988 (8)	0.0312 (3)	
N1	1.09012 (11)	0.98349 (10)	0.29628 (9)	0.0348 (3)	
N4	1.38732 (10)	1.53634 (10)	−0.21601 (9)	0.0328 (3)	
N2	0.85504 (11)	1.04057 (10)	0.31760 (9)	0.0348 (3)	
C30	1.77665 (14)	1.14853 (13)	0.02428 (11)	0.0394 (4)	
H30	1.804805	1.210493	0.021797	0.047*	
C29	1.66650 (13)	1.14509 (12)	0.03677 (10)	0.0342 (3)	
C6	1.02902 (12)	0.72982 (11)	0.55470 (11)	0.0323 (3)	
C7	1.18525 (12)	0.64072 (12)	0.50044 (10)	0.0331 (3)	
C23	1.42594 (12)	1.29204 (11)	−0.02010 (11)	0.0323 (3)	
C34	1.62520 (15)	1.05536 (13)	0.03816 (12)	0.0418 (4)	
H34	1.549609	1.053403	0.045235	0.050*	
C1	0.94559 (13)	0.79349 (11)	0.49555 (10)	0.0331 (3)	
C2	0.84446 (13)	0.79020 (12)	0.53936 (11)	0.0365 (3)	
H2	0.782216	0.831558	0.504107	0.044*	
C28	1.49065 (12)	1.23629 (11)	0.05651 (11)	0.0337 (3)	
C5	1.01979 (14)	0.66945 (12)	0.64606 (11)	0.0384 (4)	
H5	1.082053	0.628626	0.681577	0.046*	
C3	0.82873 (14)	0.73114 (12)	0.63013 (12)	0.0395 (4)	
H3	0.757887	0.732543	0.654863	0.047*	
C27	1.45658 (15)	1.18517 (12)	0.14444 (12)	0.0428 (4)	
H27	1.507545	1.149110	0.193071	0.051*	
C42	1.64438 (13)	1.48109 (12)	−0.07123 (11)	0.0366 (3)	
H42A	1.669490	1.409884	−0.027826	0.055*	
H42B	1.578592	1.513524	−0.048071	0.055*	
H42C	1.701116	1.525264	−0.078448	0.055*	
C12	1.13614 (14)	0.55260 (12)	0.50975 (11)	0.0387 (4)	
H12	1.063499	0.551522	0.530254	0.046*	
C24	1.31489 (13)	1.29086 (12)	0.00036 (12)	0.0375 (4)	
H24	1.263632	1.326381	−0.048140	0.045*	
C17	1.12073 (14)	0.97764 (13)	0.20586 (11)	0.0395 (4)	
H17A	1.167198	1.030906	0.176246	0.059*	
H17B	1.055210	0.991561	0.168298	0.059*	
H17C	1.160271	0.906531	0.212522	0.059*	
C4	0.91735 (15)	0.67027 (12)	0.68414 (11)	0.0410 (4)	
H4	0.908137	0.629649	0.746377	0.049*	
C35	1.60971 (13)	1.22407 (12)	−0.21664 (12)	0.0391 (4)	
H35C	1.649159	1.222013	−0.161917	0.047*	0.66
H35D	1.660940	1.231984	−0.268003	0.047*	0.66
H35A	1.627592	1.199628	−0.150480	0.047*	0.34
H35B	1.674817	1.245622	−0.251879	0.047*	0.34
C41	1.72199 (13)	1.43302 (13)	−0.19669 (12)	0.0387 (4)	
H41A	1.772863	1.483505	−0.206105	0.058*	
H41B	1.707052	1.424906	−0.255199	0.058*	
H41C	1.753905	1.363923	−0.153363	0.058*	
C8	1.29127 (14)	0.64204 (14)	0.46917 (11)	0.0394 (4)	
H8	1.325211	0.702280	0.461435	0.047*	
C46	1.28302 (13)	1.55103 (13)	−0.17203 (11)	0.0385 (4)	
H46A	1.248292	1.625462	−0.197643	0.058*	
H46B	1.294731	1.532509	−0.106073	0.058*	
H46C	1.236100	1.504957	−0.182887	0.058*	
C9	1.34694 (16)	0.55517 (16)	0.44944 (12)	0.0497 (4)	
H9	1.419391	0.555980	0.428408	0.060*	
C45	1.36762 (15)	1.55682 (13)	−0.31317 (11)	0.0411 (4)	
H45A	1.319644	1.509253	−0.319713	0.062*	
H45B	1.436754	1.544013	−0.342887	0.062*	
H45C	1.333274	1.630901	−0.342007	0.062*	
C44	1.45599 (13)	1.61023 (12)	−0.20422 (11)	0.0364 (3)	
H44A	1.449918	1.610992	−0.140674	0.044*	
H44B	1.429330	1.683073	−0.245888	0.044*	
C43	1.57392 (13)	1.57864 (12)	−0.22404 (11)	0.0362 (3)	
H43A	1.579953	1.577318	−0.287423	0.043*	
H43B	1.616120	1.632463	−0.219290	0.043*	
C38	1.42701 (15)	1.27399 (15)	−0.26408 (15)	0.0506 (5)	
H38C	1.396498	1.321975	−0.324324	0.061*	0.66
H38D	1.369799	1.271314	−0.217660	0.061*	0.66
H38A	1.417150	1.297834	−0.331331	0.061*	0.34
H38B	1.359992	1.300654	−0.237093	0.061*	0.34
C15	0.87567 (15)	0.66987 (14)	0.25288 (14)	0.0488 (4)	
H15A	0.868592	0.682091	0.186679	0.059*	
H15B	0.829120	0.617537	0.287298	0.059*	
C25	1.27537 (14)	1.24144 (12)	0.08637 (13)	0.0436 (4)	
H25	1.199380	1.244176	0.095192	0.052*	
C11	1.19251 (17)	0.46675 (14)	0.48933 (12)	0.0487 (4)	
H11	1.158209	0.407103	0.495553	0.058*	
C18	1.18808 (14)	0.95541 (15)	0.35355 (12)	0.0460 (4)	
H18A	1.223014	0.882972	0.359505	0.069*	
H18B	1.168471	0.959149	0.414268	0.069*	
H18C	1.238360	1.005292	0.325239	0.069*	
C10	1.29811 (19)	0.46684 (15)	0.46001 (13)	0.0549 (5)	
H10	1.337123	0.407054	0.447152	0.066*	
C26	1.34627 (16)	1.18844 (13)	0.15890 (13)	0.0467 (4)	
H26	1.319815	1.154629	0.218013	0.056*	
C13	1.03659 (14)	0.73595 (13)	0.26162 (13)	0.0426 (4)	
H13A	1.095689	0.729181	0.304784	0.051*	
H13B	1.065423	0.756004	0.198776	0.051*	
C31	1.84516 (16)	1.06123 (16)	0.01548 (13)	0.0509 (4)	
H31	1.920636	1.063261	0.007283	0.061*	
C16	0.84742 (14)	0.77276 (14)	0.27319 (14)	0.0442 (4)	
H16A	0.793938	0.823757	0.227668	0.053*	
H16B	0.816019	0.759489	0.334893	0.053*	
C22	0.75391 (14)	1.04273 (13)	0.27195 (12)	0.0418 (4)	
H22A	0.712989	1.115157	0.252207	0.063*	
H22B	0.710298	0.994274	0.314597	0.063*	
H22C	0.770819	1.020210	0.218532	0.063*	
C20	0.92107 (14)	1.11342 (12)	0.25394 (11)	0.0399 (4)	
H20A	0.889002	1.187751	0.246874	0.048*	
H20B	0.919870	1.104868	0.193146	0.048*	
C21	0.82983 (15)	1.07237 (14)	0.39835 (12)	0.0439 (4)	
H21A	0.789870	1.145175	0.379448	0.066*	
H21B	0.897438	1.069211	0.429694	0.066*	
H21C	0.785305	1.024005	0.439968	0.066*	
C19	1.03724 (14)	1.09228 (12)	0.28750 (12)	0.0396 (4)	
H19A	1.078265	1.144139	0.244120	0.048*	
H19B	1.038598	1.102323	0.347691	0.048*	
C33	1.69527 (18)	0.96858 (15)	0.02912 (15)	0.0562 (5)	
H33	1.667240	0.907010	0.030291	0.067*	
C32	1.80453 (19)	0.97066 (16)	0.01851 (15)	0.0600 (5)	
H32	1.852047	0.910503	0.013283	0.072*	
C14	0.99292 (15)	0.63327 (15)	0.28526 (17)	0.0569 (5)	
H14A	0.997947	0.594350	0.351941	0.068*	
H14B	1.032292	0.587014	0.252712	0.068*	
Li1	0.9638 (2)	0.8908 (2)	0.35758 (17)	0.0347 (5)	
Li2	1.4880 (2)	1.38462 (19)	−0.14696 (18)	0.0327 (5)	
C37	1.4691 (4)	1.1615 (4)	−0.2676 (3)	0.0545 (11)	0.66
H37A	1.412378	1.115409	−0.249820	0.065*	0.66
H37B	1.495830	1.164060	−0.329146	0.065*	0.66
C36	1.5606 (4)	1.1245 (3)	−0.1976 (3)	0.0487 (9)	0.66
H36A	1.532973	1.100422	−0.134463	0.058*	0.66
H36B	1.613702	1.066287	−0.207117	0.058*	0.66
C40	1.5684 (8)	1.1354 (7)	−0.2431 (6)	0.054 (2)	0.34
H40A	1.582591	1.142537	−0.307938	0.065*	0.34
H40B	1.600727	1.063315	−0.203265	0.065*	0.34
C39	1.4523 (9)	1.1596 (9)	−0.2264 (9)	0.074 (3)	0.34
H39A	1.409276	1.125986	−0.257567	0.089*	0.34
H39B	1.437392	1.134166	−0.160125	0.089*	0.34

### (2-phenoxyphenyl-κC1)(tetrahydrofuran-κO)(N,N,N',N'-tetramethylethylenediamine-κ2N,N')lithium (1) Atomic displacement parameters (Å^2^)

**Table:**

	*U*^11^	*U*^22^	*U*^33^	*U*^12^	*U*^13^	*U*^23^
O4	0.0312 (5)	0.0365 (5)	0.0400 (6)	−0.0036 (4)	0.0011 (4)	−0.0188 (5)
O2	0.0333 (6)	0.0377 (6)	0.0413 (6)	−0.0087 (4)	0.0011 (5)	−0.0183 (5)
O1	0.0315 (6)	0.0320 (5)	0.0570 (7)	−0.0096 (4)	0.0055 (5)	−0.0152 (5)
O3	0.0288 (6)	0.0285 (5)	0.0652 (8)	−0.0040 (4)	−0.0011 (5)	−0.0146 (5)
N3	0.0307 (6)	0.0313 (6)	0.0325 (7)	−0.0067 (5)	0.0035 (5)	−0.0116 (5)
N1	0.0367 (7)	0.0351 (6)	0.0324 (7)	−0.0119 (5)	0.0012 (5)	−0.0085 (5)
N4	0.0322 (7)	0.0333 (6)	0.0328 (7)	−0.0041 (5)	−0.0012 (5)	−0.0112 (5)
N2	0.0380 (7)	0.0328 (6)	0.0345 (7)	−0.0069 (5)	0.0019 (6)	−0.0119 (5)
C30	0.0381 (9)	0.0443 (9)	0.0319 (8)	−0.0044 (7)	−0.0003 (7)	−0.0084 (7)
C29	0.0365 (8)	0.0322 (7)	0.0290 (8)	−0.0007 (6)	−0.0008 (6)	−0.0063 (6)
C6	0.0329 (8)	0.0267 (7)	0.0396 (8)	−0.0099 (6)	0.0037 (6)	−0.0122 (6)
C7	0.0341 (8)	0.0342 (7)	0.0280 (7)	−0.0026 (6)	−0.0041 (6)	−0.0073 (6)
C23	0.0319 (8)	0.0265 (7)	0.0415 (8)	−0.0072 (6)	0.0045 (6)	−0.0143 (6)
C34	0.0469 (10)	0.0351 (8)	0.0430 (9)	−0.0067 (7)	0.0062 (7)	−0.0132 (7)
C1	0.0359 (8)	0.0297 (7)	0.0348 (8)	−0.0092 (6)	0.0011 (6)	−0.0107 (6)
C2	0.0343 (8)	0.0327 (7)	0.0432 (9)	−0.0078 (6)	−0.0002 (7)	−0.0122 (6)
C28	0.0311 (8)	0.0251 (7)	0.0472 (9)	−0.0083 (6)	0.0048 (7)	−0.0137 (6)
C5	0.0442 (9)	0.0287 (7)	0.0399 (9)	−0.0087 (6)	−0.0071 (7)	−0.0061 (6)
C3	0.0412 (9)	0.0362 (8)	0.0470 (9)	−0.0157 (7)	0.0122 (7)	−0.0185 (7)
C27	0.0535 (10)	0.0314 (8)	0.0427 (9)	−0.0134 (7)	0.0001 (8)	−0.0082 (7)
C42	0.0373 (8)	0.0379 (8)	0.0382 (9)	−0.0116 (7)	0.0014 (7)	−0.0148 (7)
C12	0.0442 (9)	0.0359 (8)	0.0355 (8)	−0.0086 (7)	0.0013 (7)	−0.0105 (6)
C24	0.0322 (8)	0.0299 (7)	0.0520 (10)	−0.0071 (6)	0.0032 (7)	−0.0152 (7)
C17	0.0416 (9)	0.0399 (8)	0.0387 (9)	−0.0132 (7)	0.0058 (7)	−0.0129 (7)
C4	0.0569 (11)	0.0330 (8)	0.0345 (8)	−0.0189 (7)	0.0065 (7)	−0.0084 (6)
C35	0.0352 (8)	0.0356 (8)	0.0449 (9)	−0.0034 (7)	0.0010 (7)	−0.0125 (7)
C41	0.0315 (8)	0.0412 (8)	0.0446 (9)	−0.0073 (7)	0.0069 (7)	−0.0161 (7)
C8	0.0384 (9)	0.0473 (9)	0.0282 (8)	−0.0066 (7)	−0.0001 (7)	−0.0069 (7)
C46	0.0308 (8)	0.0435 (8)	0.0394 (9)	0.0005 (7)	−0.0027 (7)	−0.0143 (7)
C9	0.0458 (10)	0.0634 (11)	0.0314 (9)	0.0024 (9)	0.0080 (7)	−0.0110 (8)
C45	0.0477 (10)	0.0407 (8)	0.0343 (8)	−0.0032 (7)	−0.0058 (7)	−0.0130 (7)
C44	0.0401 (9)	0.0307 (7)	0.0395 (9)	−0.0050 (6)	−0.0016 (7)	−0.0132 (6)
C43	0.0384 (8)	0.0314 (7)	0.0384 (8)	−0.0109 (6)	0.0038 (7)	−0.0089 (6)
C38	0.0397 (10)	0.0513 (10)	0.0698 (13)	−0.0061 (8)	−0.0094 (9)	−0.0315 (9)
C15	0.0438 (10)	0.0445 (9)	0.0641 (12)	−0.0153 (8)	−0.0021 (8)	−0.0219 (8)
C25	0.0363 (9)	0.0342 (8)	0.0647 (12)	−0.0134 (7)	0.0183 (8)	−0.0208 (8)
C11	0.0688 (13)	0.0387 (9)	0.0402 (9)	−0.0087 (8)	0.0089 (9)	−0.0160 (7)
C18	0.0406 (9)	0.0550 (10)	0.0395 (9)	−0.0185 (8)	−0.0040 (7)	−0.0064 (8)
C10	0.0716 (14)	0.0474 (10)	0.0407 (10)	0.0030 (9)	0.0126 (9)	−0.0158 (8)
C26	0.0601 (11)	0.0365 (8)	0.0470 (10)	−0.0214 (8)	0.0197 (9)	−0.0142 (7)
C13	0.0354 (9)	0.0397 (8)	0.0554 (11)	−0.0068 (7)	0.0004 (7)	−0.0189 (8)
C31	0.0413 (10)	0.0638 (11)	0.0403 (10)	0.0051 (9)	0.0073 (8)	−0.0151 (8)
C16	0.0328 (8)	0.0451 (9)	0.0582 (11)	−0.0106 (7)	−0.0003 (8)	−0.0196 (8)
C22	0.0388 (9)	0.0390 (8)	0.0442 (9)	−0.0027 (7)	−0.0042 (7)	−0.0107 (7)
C20	0.0474 (10)	0.0293 (7)	0.0389 (9)	−0.0056 (7)	0.0056 (7)	−0.0071 (6)
C21	0.0509 (10)	0.0442 (9)	0.0422 (9)	−0.0130 (8)	0.0101 (8)	−0.0204 (7)
C19	0.0465 (9)	0.0333 (8)	0.0429 (9)	−0.0163 (7)	0.0078 (7)	−0.0140 (7)
C33	0.0678 (13)	0.0413 (9)	0.0622 (12)	−0.0044 (9)	0.0133 (10)	−0.0242 (9)
C32	0.0705 (14)	0.0502 (11)	0.0558 (12)	0.0069 (10)	0.0136 (10)	−0.0225 (9)
C14	0.0410 (10)	0.0382 (9)	0.0897 (16)	−0.0087 (8)	0.0019 (10)	−0.0182 (9)
Li1	0.0376 (14)	0.0347 (12)	0.0322 (13)	−0.0088 (11)	0.0018 (11)	−0.0106 (10)
Li2	0.0315 (13)	0.0328 (12)	0.0350 (13)	−0.0054 (10)	0.0029 (10)	−0.0132 (10)
C37	0.051 (3)	0.0548 (19)	0.075 (3)	−0.0118 (18)	−0.001 (2)	−0.044 (2)
C36	0.0414 (18)	0.0363 (15)	0.067 (3)	−0.0082 (12)	−0.002 (2)	−0.014 (2)
C40	0.059 (5)	0.035 (3)	0.073 (6)	−0.001 (3)	0.001 (5)	−0.026 (5)
C39	0.052 (4)	0.062 (5)	0.134 (10)	−0.021 (3)	0.030 (7)	−0.065 (7)

### (2-phenoxyphenyl-κC1)(tetrahydrofuran-κO)(N,N,N',N'-tetramethylethylenediamine-κ2N,N')lithium (1) Geometric parameters (Å, º)

**Table:**

O4—C35	1.4470 (19)	C41—H41C	0.9800
O4—C38	1.4390 (19)	C8—H8	0.9500
O4—Li2	2.009 (3)	C8—C9	1.384 (3)
O2—C13	1.436 (2)	C46—H46A	0.9800
O2—C16	1.4380 (18)	C46—H46B	0.9800
O2—Li1	2.023 (3)	C46—H46C	0.9800
O1—C6	1.4290 (19)	C9—H9	0.9500
O1—C7	1.3625 (19)	C9—C10	1.388 (3)
O3—C29	1.3681 (19)	C45—H45A	0.9800
O3—C28	1.4293 (18)	C45—H45B	0.9800
N3—C42	1.4640 (19)	C45—H45C	0.9800
N3—C41	1.467 (2)	C44—H44A	0.9900
N3—C43	1.478 (2)	C44—H44B	0.9900
N3—Li2	2.162 (3)	C44—C43	1.514 (2)
N1—C17	1.464 (2)	C43—H43A	0.9900
N1—C18	1.464 (2)	C43—H43B	0.9900
N1—C19	1.474 (2)	C38—H38C	0.9900
N1—Li1	2.167 (3)	C38—H38D	0.9900
N4—C46	1.462 (2)	C38—H38A	0.9900
N4—C45	1.463 (2)	C38—H38B	0.9900
N4—C44	1.4795 (19)	C38—C37	1.545 (5)
N4—Li2	2.172 (3)	C38—C39	1.442 (12)
N2—C22	1.466 (2)	C15—H15A	0.9900
N2—C20	1.474 (2)	C15—H15B	0.9900
N2—C21	1.464 (2)	C15—C16	1.512 (2)
N2—Li1	2.156 (3)	C15—C14	1.516 (3)
C30—H30	0.9500	C25—H25	0.9500
C30—C29	1.388 (2)	C25—C26	1.377 (3)
C30—C31	1.383 (3)	C11—H11	0.9500
C29—C34	1.389 (2)	C11—C10	1.378 (3)
C6—C1	1.386 (2)	C18—H18A	0.9800
C6—C5	1.389 (2)	C18—H18B	0.9800
C7—C12	1.392 (2)	C18—H18C	0.9800
C7—C8	1.393 (2)	C10—H10	0.9500
C23—C28	1.382 (2)	C26—H26	0.9500
C23—C24	1.412 (2)	C13—H13A	0.9900
C23—Li2	2.140 (3)	C13—H13B	0.9900
C34—H34	0.9500	C13—C14	1.501 (2)
C34—C33	1.388 (3)	C31—H31	0.9500
C1—C2	1.410 (2)	C31—C32	1.388 (3)
C1—Li1	2.129 (3)	C16—H16A	0.9900
C2—H2	0.9500	C16—H16B	0.9900
C2—C3	1.390 (2)	C22—H22A	0.9800
C28—C27	1.391 (2)	C22—H22B	0.9800
C5—H5	0.9500	C22—H22C	0.9800
C5—C4	1.387 (2)	C20—H20A	0.9900
C3—H3	0.9500	C20—H20B	0.9900
C3—C4	1.385 (3)	C20—C19	1.512 (2)
C27—H27	0.9500	C21—H21A	0.9800
C27—C26	1.383 (3)	C21—H21B	0.9800
C42—H42A	0.9800	C21—H21C	0.9800
C42—H42B	0.9800	C19—H19A	0.9900
C42—H42C	0.9800	C19—H19B	0.9900
C12—H12	0.9500	C33—H33	0.9500
C12—C11	1.380 (2)	C33—C32	1.372 (3)
C24—H24	0.9500	C32—H32	0.9500
C24—C25	1.388 (2)	C14—H14A	0.9900
C17—H17A	0.9800	C14—H14B	0.9900
C17—H17B	0.9800	C37—H37A	0.9900
C17—H17C	0.9800	C37—H37B	0.9900
C4—H4	0.9500	C37—C36	1.513 (7)
C35—H35C	0.9900	C36—H36A	0.9900
C35—H35D	0.9900	C36—H36B	0.9900
C35—H35A	0.9900	C40—H40A	0.9900
C35—H35B	0.9900	C40—H40B	0.9900
C35—C36	1.505 (4)	C40—C39	1.465 (15)
C35—C40	1.559 (8)	C39—H39A	0.9900
C41—H41A	0.9800	C39—H39B	0.9900
C41—H41B	0.9800		
			
C35—O4—Li2	114.72 (12)	C43—C44—H44B	109.2
C38—O4—C35	108.76 (12)	N3—C43—C44	111.76 (12)
C38—O4—Li2	113.98 (13)	N3—C43—H43A	109.3
C13—O2—C16	109.44 (12)	N3—C43—H43B	109.3
C13—O2—Li1	117.11 (12)	C44—C43—H43A	109.3
C16—O2—Li1	116.08 (13)	C44—C43—H43B	109.3
C7—O1—C6	118.30 (11)	H43A—C43—H43B	107.9
C29—O3—C28	117.95 (11)	O4—C38—H38C	110.5
C42—N3—C41	108.37 (12)	O4—C38—H38D	110.5
C42—N3—C43	110.21 (12)	O4—C38—H38A	110.8
C42—N3—Li2	109.94 (11)	O4—C38—H38B	110.8
C41—N3—C43	109.44 (12)	O4—C38—C37	106.1 (2)
C41—N3—Li2	116.74 (11)	O4—C38—C39	104.6 (5)
C43—N3—Li2	101.95 (11)	H38C—C38—H38D	108.7
C17—N1—C19	109.86 (13)	H38A—C38—H38B	108.9
C17—N1—Li1	112.69 (11)	C37—C38—H38C	110.5
C18—N1—C17	108.82 (14)	C37—C38—H38D	110.5
C18—N1—C19	109.57 (13)	C39—C38—H38A	110.8
C18—N1—Li1	113.65 (12)	C39—C38—H38B	110.8
C19—N1—Li1	102.04 (12)	H15A—C15—H15B	109.2
C46—N4—C45	108.69 (13)	C16—C15—H15A	111.3
C46—N4—C44	109.57 (12)	C16—C15—H15B	111.3
C46—N4—Li2	113.27 (12)	C16—C15—C14	102.27 (14)
C45—N4—C44	110.53 (12)	C14—C15—H15A	111.3
C45—N4—Li2	114.14 (12)	C14—C15—H15B	111.3
C44—N4—Li2	100.35 (11)	C24—C25—H25	120.0
C22—N2—C20	109.81 (13)	C26—C25—C24	120.01 (16)
C22—N2—Li1	115.66 (12)	C26—C25—H25	120.0
C20—N2—Li1	102.67 (12)	C12—C11—H11	119.7
C21—N2—C22	109.51 (13)	C10—C11—C12	120.55 (17)
C21—N2—C20	110.03 (12)	C10—C11—H11	119.7
C21—N2—Li1	108.93 (12)	N1—C18—H18A	109.5
C29—C30—H30	120.2	N1—C18—H18B	109.5
C31—C30—H30	120.2	N1—C18—H18C	109.5
C31—C30—C29	119.60 (16)	H18A—C18—H18B	109.5
O3—C29—C30	115.66 (14)	H18A—C18—H18C	109.5
O3—C29—C34	124.28 (15)	H18B—C18—H18C	109.5
C30—C29—C34	120.05 (15)	C9—C10—H10	120.3
C1—C6—O1	117.51 (13)	C11—C10—C9	119.35 (17)
C1—C6—C5	126.83 (15)	C11—C10—H10	120.3
C5—C6—O1	115.61 (14)	C27—C26—H26	120.5
O1—C7—C12	124.56 (15)	C25—C26—C27	119.02 (16)
O1—C7—C8	116.07 (13)	C25—C26—H26	120.5
C12—C7—C8	119.36 (15)	O2—C13—H13A	110.5
C28—C23—C24	111.21 (14)	O2—C13—H13B	110.5
C28—C23—Li2	123.09 (13)	O2—C13—C14	106.20 (14)
C24—C23—Li2	125.20 (14)	H13A—C13—H13B	108.7
C29—C34—H34	120.3	C14—C13—H13A	110.5
C33—C34—C29	119.46 (17)	C14—C13—H13B	110.5
C33—C34—H34	120.3	C30—C31—H31	119.7
C6—C1—C2	111.36 (14)	C30—C31—C32	120.52 (18)
C6—C1—Li1	125.77 (14)	C32—C31—H31	119.7
C2—C1—Li1	122.82 (14)	O2—C16—C15	106.47 (14)
C1—C2—H2	117.5	O2—C16—H16A	110.4
C3—C2—C1	125.09 (15)	O2—C16—H16B	110.4
C3—C2—H2	117.5	C15—C16—H16A	110.4
C23—C28—O3	117.84 (14)	C15—C16—H16B	110.4
C23—C28—C27	127.18 (15)	H16A—C16—H16B	108.6
C27—C28—O3	114.91 (14)	N2—C22—H22A	109.5
C6—C5—H5	120.8	N2—C22—H22B	109.5
C4—C5—C6	118.32 (15)	N2—C22—H22C	109.5
C4—C5—H5	120.8	H22A—C22—H22B	109.5
C2—C3—H3	120.3	H22A—C22—H22C	109.5
C4—C3—C2	119.35 (16)	H22B—C22—H22C	109.5
C4—C3—H3	120.3	N2—C20—H20A	109.3
C28—C27—H27	121.0	N2—C20—H20B	109.3
C26—C27—C28	117.99 (16)	N2—C20—C19	111.71 (13)
C26—C27—H27	121.0	H20A—C20—H20B	107.9
N3—C42—H42A	109.5	C19—C20—H20A	109.3
N3—C42—H42B	109.5	C19—C20—H20B	109.3
N3—C42—H42C	109.5	N2—C21—H21A	109.5
H42A—C42—H42B	109.5	N2—C21—H21B	109.5
H42A—C42—H42C	109.5	N2—C21—H21C	109.5
H42B—C42—H42C	109.5	H21A—C21—H21B	109.5
C7—C12—H12	119.9	H21A—C21—H21C	109.5
C11—C12—C7	120.29 (17)	H21B—C21—H21C	109.5
C11—C12—H12	119.9	N1—C19—C20	111.53 (12)
C23—C24—H24	117.7	N1—C19—H19A	109.3
C25—C24—C23	124.58 (16)	N1—C19—H19B	109.3
C25—C24—H24	117.7	C20—C19—H19A	109.3
N1—C17—H17A	109.5	C20—C19—H19B	109.3
N1—C17—H17B	109.5	H19A—C19—H19B	108.0
N1—C17—H17C	109.5	C34—C33—H33	119.6
H17A—C17—H17B	109.5	C32—C33—C34	120.78 (18)
H17A—C17—H17C	109.5	C32—C33—H33	119.6
H17B—C17—H17C	109.5	C31—C32—H32	120.2
C5—C4—H4	120.5	C33—C32—C31	119.55 (18)
C3—C4—C5	119.04 (15)	C33—C32—H32	120.2
C3—C4—H4	120.5	C15—C14—H14A	111.2
O4—C35—H35C	110.6	C15—C14—H14B	111.2
O4—C35—H35D	110.6	C13—C14—C15	102.81 (15)
O4—C35—H35A	111.0	C13—C14—H14A	111.2
O4—C35—H35B	111.0	C13—C14—H14B	111.2
O4—C35—C36	105.6 (2)	H14A—C14—H14B	109.1
O4—C35—C40	103.9 (4)	O2—Li1—N1	102.62 (12)
H35C—C35—H35D	108.7	O2—Li1—N2	109.23 (12)
H35A—C35—H35B	109.0	O2—Li1—C1	111.96 (12)
C36—C35—H35C	110.6	N2—Li1—N1	85.91 (10)
C36—C35—H35D	110.6	C1—Li1—N1	129.95 (13)
C40—C35—H35A	111.0	C1—Li1—N2	113.70 (13)
C40—C35—H35B	111.0	O4—Li2—N3	104.24 (12)
N3—C41—H41A	109.5	O4—Li2—N4	106.42 (12)
N3—C41—H41B	109.5	O4—Li2—C23	115.17 (12)
N3—C41—H41C	109.5	N3—Li2—N4	86.74 (10)
H41A—C41—H41B	109.5	C23—Li2—N3	124.95 (13)
H41A—C41—H41C	109.5	C23—Li2—N4	114.98 (13)
H41B—C41—H41C	109.5	C38—C37—H37A	111.6
C7—C8—H8	120.2	C38—C37—H37B	111.6
C9—C8—C7	119.68 (16)	H37A—C37—H37B	109.4
C9—C8—H8	120.2	C36—C37—C38	100.9 (3)
N4—C46—H46A	109.5	C36—C37—H37A	111.6
N4—C46—H46B	109.5	C36—C37—H37B	111.6
N4—C46—H46C	109.5	C35—C36—C37	101.8 (3)
H46A—C46—H46B	109.5	C35—C36—H36A	111.4
H46A—C46—H46C	109.5	C35—C36—H36B	111.4
H46B—C46—H46C	109.5	C37—C36—H36A	111.4
C8—C9—H9	119.6	C37—C36—H36B	111.4
C8—C9—C10	120.75 (18)	H36A—C36—H36B	109.3
C10—C9—H9	119.6	C35—C40—H40A	111.9
N4—C45—H45A	109.5	C35—C40—H40B	111.9
N4—C45—H45B	109.5	H40A—C40—H40B	109.6
N4—C45—H45C	109.5	C39—C40—C35	99.5 (6)
H45A—C45—H45B	109.5	C39—C40—H40A	111.9
H45A—C45—H45C	109.5	C39—C40—H40B	111.9
H45B—C45—H45C	109.5	C38—C39—C40	104.5 (8)
N4—C44—H44A	109.2	C38—C39—H39A	110.9
N4—C44—H44B	109.2	C38—C39—H39B	110.9
N4—C44—C43	111.85 (12)	C40—C39—H39A	110.9
H44A—C44—H44B	107.9	C40—C39—H39B	110.9
C43—C44—H44A	109.2	H39A—C39—H39B	108.9
			
O4—C35—C36—C37	36.6 (3)	C12—C7—C8—C9	−1.2 (2)
O4—C35—C40—C39	−30.9 (8)	C12—C11—C10—C9	−1.2 (3)
O4—C38—C37—C36	30.0 (4)	C24—C23—C28—O3	−177.65 (11)
O4—C38—C39—C40	−39.3 (8)	C24—C23—C28—C27	−0.9 (2)
O2—C13—C14—C15	30.7 (2)	C24—C25—C26—C27	−0.2 (2)
O1—C6—C1—C2	−177.94 (12)	C17—N1—C19—C20	77.42 (16)
O1—C6—C1—Li1	−0.6 (2)	C35—O4—C38—C37	−7.8 (3)
O1—C6—C5—C4	178.14 (12)	C35—O4—C38—C39	18.2 (5)
O1—C7—C12—C11	−178.86 (15)	C35—C40—C39—C38	42.7 (9)
O1—C7—C8—C9	178.55 (14)	C41—N3—C43—C44	163.99 (12)
O3—C29—C34—C33	−178.20 (16)	C8—C7—C12—C11	0.9 (2)
O3—C28—C27—C26	177.31 (13)	C8—C9—C10—C11	0.9 (3)
N4—C44—C43—N3	−62.57 (17)	C46—N4—C44—C43	163.97 (13)
N2—C20—C19—N1	60.85 (18)	C45—N4—C44—C43	−76.28 (16)
C30—C29—C34—C33	1.5 (3)	C38—O4—C35—C36	−18.0 (2)
C30—C31—C32—C33	0.9 (3)	C38—O4—C35—C40	8.3 (4)
C29—O3—C28—C23	−99.69 (16)	C38—C37—C36—C35	−39.7 (4)
C29—O3—C28—C27	83.16 (17)	C18—N1—C19—C20	−163.08 (13)
C29—C30—C31—C32	0.4 (3)	C13—O2—C16—C15	−9.46 (19)
C29—C34—C33—C32	−0.2 (3)	C31—C30—C29—O3	178.16 (15)
C6—O1—C7—C12	1.2 (2)	C31—C30—C29—C34	−1.6 (2)
C6—O1—C7—C8	−178.56 (13)	C16—O2—C13—C14	−13.52 (19)
C6—C1—C2—C3	0.0 (2)	C16—C15—C14—C13	−35.3 (2)
C6—C5—C4—C3	−0.2 (2)	C22—N2—C20—C19	−164.24 (13)
C7—O1—C6—C1	−105.52 (15)	C21—N2—C20—C19	75.15 (17)
C7—O1—C6—C5	76.77 (17)	C14—C15—C16—O2	28.0 (2)
C7—C12—C11—C10	0.4 (3)	Li1—O2—C13—C14	121.27 (16)
C7—C8—C9—C10	0.3 (3)	Li1—O2—C16—C15	−144.77 (14)
C23—C28—C27—C26	0.5 (2)	Li1—N1—C19—C20	−42.34 (16)
C23—C24—C25—C26	−0.3 (2)	Li1—N2—C20—C19	−40.69 (16)
C34—C33—C32—C31	−0.9 (3)	Li1—C1—C2—C3	−177.51 (14)
C1—C6—C5—C4	0.7 (2)	Li2—O4—C35—C36	111.0 (2)
C1—C2—C3—C4	0.4 (2)	Li2—O4—C35—C40	137.3 (4)
C2—C3—C4—C5	−0.3 (2)	Li2—O4—C38—C37	−137.2 (2)
C28—O3—C29—C30	177.26 (14)	Li2—O4—C38—C39	−111.1 (5)
C28—O3—C29—C34	−3.0 (2)	Li2—N3—C43—C44	39.77 (15)
C28—C23—C24—C25	0.8 (2)	Li2—N4—C44—C43	44.55 (15)
C28—C27—C26—C25	0.1 (2)	Li2—C23—C28—O3	−5.38 (19)
C5—C6—C1—C2	−0.5 (2)	Li2—C23—C28—C27	171.38 (14)
C5—C6—C1—Li1	176.85 (14)	Li2—C23—C24—C25	−171.28 (14)
C42—N3—C43—C44	−76.93 (15)		

### (2-phenoxyphenyl-κC1)(tetrahydrofuran-κO)(N,N,N',N'-tetramethylethylenediamine-κ2N,N')lithium (1) Hydrogen-bond geometry (Å, º)

**Table:**

*D*—H···*A*	*D*—H	H···*A*	*D*···*A*	*D*—H···*A*
C9—H9···O1	0.95	3.02	3.612 (2)	124

### (2) Crystal data

**Table:**

C_12_H_10_O	*D*_x_ = 1.249 Mg m^−^^3^
*M**_r_* = 170.20	Mo *K*α radiation, λ = 0.71073 Å
Orthorhombic, *P*2_1_2_1_2_1_	Cell parameters from 237 reflections
*a* = 5.6154 (10) Å	θ = 2.8–21.6°
*b* = 7.7194 (11) Å	µ = 0.08 mm^−^^1^
*c* = 20.884 (3) Å	*T* = 100 K
*V* = 905.3 (2) Å^3^	Plate, colourless
*Z* = 4	0.94 × 0.72 × 0.17 mm
*F*(000) = 360	

### (2) Data collection

**Table:**

Bruker APEXII CCD diffractometer	3439 independent reflections
Radiation source: microfocus sealed X-ray tube, Incoatec Iµs	3113 reflections with *I* > 2σ(*I*)
HELIOS mirror optics monochromator	*R*_int_ = 0.058
Detector resolution: 10.4167 pixels mm^-1^	θ_max_ = 33.2°, θ_min_ = 2.0°
φ and ω scans	*h* = −8→8
Absorption correction: multi-scan (SADABS; Krause *et al.*, 2015)	*k* = −11→11
*T*_min_ = 0.487, *T*_max_ = 0.566	*l* = −32→31
29791 measured reflections	

### (2) Refinement

**Table:**

Refinement on *F*^2^	Hydrogen site location: difference Fourier map
Least-squares matrix: full	All H-atom parameters refined
*R*[*F*^2^ > 2σ(*F*^2^)] = 0.039	*w* = 1/[σ^2^(*F*_o_^2^) + (0.0636*P*)^2^ + 0.0911*P*] where *P* = (*F*_o_^2^ + 2*F*_c_^2^)/3
*wR*(*F*^2^) = 0.109	(Δ/σ)_max_ = 0.001
*S* = 1.06	Δρ_max_ = 0.33 e Å^−^^3^
3439 reflections	Δρ_min_ = −0.18 e Å^−^^3^
158 parameters	Absolute structure: Flack *x* determined using 1169 quotients [(*I*^+^)-(*I*^-^)]/[(*I*^+^)+(*I*^-^)] (Parsons et al., 2013)
0 restraints	Absolute structure parameter: −0.8 (5)
Primary atom site location: dual	

### (2) Special details

**Table:**

Geometry. All esds (except the esd in the dihedral angle between two l.s. planes) are estimated using the full covariance matrix. The cell esds are taken into account individually in the estimation of esds in distances, angles and torsion angles; correlations between esds in cell parameters are only used when they are defined by crystal symmetry. An approximate (isotropic) treatment of cell esds is used for estimating esds involving l.s. planes.

### (2) Fractional atomic coordinates and isotropic or equivalent isotropic displacement parameters (Å^2^)

**Table:**

	*x*	*y*	*z*	*U*_iso_*/*U*_eq_	
O1	0.12721 (19)	0.32715 (13)	0.58699 (5)	0.0226 (2)	
C7	0.1470 (2)	0.21161 (15)	0.63703 (6)	0.0166 (2)	
C2	0.3049 (2)	0.45233 (16)	0.58008 (6)	0.0179 (2)	
C8	−0.0398 (2)	0.09343 (17)	0.64271 (7)	0.0207 (2)	
C3	0.2791 (3)	0.61079 (16)	0.61061 (6)	0.0198 (2)	
C5	0.6446 (3)	0.70597 (18)	0.56067 (7)	0.0232 (3)	
C4	0.4512 (3)	0.73786 (17)	0.60055 (7)	0.0221 (3)	
C12	0.3363 (2)	0.21018 (16)	0.68004 (6)	0.0183 (2)	
C11	0.3355 (3)	0.08988 (19)	0.72985 (7)	0.0227 (3)	
C1	0.4960 (3)	0.41762 (17)	0.54011 (7)	0.0220 (3)	
C9	−0.0374 (3)	−0.02561 (19)	0.69248 (8)	0.0250 (3)	
C6	0.6667 (3)	0.54582 (19)	0.53047 (7)	0.0242 (3)	
C10	0.1494 (3)	−0.0280 (2)	0.73650 (7)	0.0265 (3)	
H3	0.138 (4)	0.630 (3)	0.6386 (11)	0.031 (6)*	
H6	0.801 (5)	0.524 (3)	0.5013 (11)	0.037 (6)*	
H5	0.758 (5)	0.790 (3)	0.5546 (11)	0.037 (6)*	
H1	0.511 (4)	0.310 (3)	0.5207 (9)	0.021 (5)*	
H10	0.147 (4)	−0.107 (3)	0.7718 (11)	0.036 (6)*	
H8	−0.172 (4)	0.097 (3)	0.6115 (10)	0.032 (6)*	
H9	−0.171 (5)	−0.101 (3)	0.6975 (11)	0.042 (7)*	
H12	0.464 (4)	0.284 (3)	0.6755 (9)	0.018 (4)*	
H11	0.461 (4)	0.089 (3)	0.7599 (11)	0.031 (6)*	
H4	0.434 (4)	0.855 (3)	0.6216 (11)	0.031 (6)*	

### (2) Atomic displacement parameters (Å^2^)

**Table:**

	*U*^11^	*U*^22^	*U*^33^	*U*^12^	*U*^13^	*U*^23^
O1	0.0245 (5)	0.0196 (4)	0.0236 (4)	−0.0074 (4)	−0.0079 (4)	0.0052 (3)
C7	0.0184 (5)	0.0142 (4)	0.0171 (5)	−0.0001 (4)	0.0010 (4)	−0.0018 (4)
C2	0.0205 (5)	0.0150 (4)	0.0181 (5)	−0.0026 (4)	−0.0033 (4)	0.0016 (4)
C8	0.0175 (5)	0.0195 (5)	0.0251 (6)	−0.0024 (4)	0.0013 (5)	−0.0008 (5)
C3	0.0216 (6)	0.0183 (5)	0.0193 (5)	−0.0004 (4)	0.0002 (5)	−0.0008 (4)
C5	0.0230 (6)	0.0195 (5)	0.0271 (6)	−0.0038 (5)	−0.0021 (5)	0.0054 (5)
C4	0.0260 (6)	0.0160 (5)	0.0243 (6)	−0.0020 (4)	−0.0028 (5)	−0.0008 (4)
C12	0.0187 (5)	0.0165 (5)	0.0197 (5)	0.0000 (4)	−0.0003 (4)	−0.0006 (4)
C11	0.0243 (6)	0.0229 (6)	0.0207 (5)	0.0021 (5)	−0.0005 (5)	0.0027 (5)
C1	0.0265 (7)	0.0172 (5)	0.0222 (6)	0.0014 (5)	0.0002 (5)	−0.0001 (4)
C9	0.0218 (6)	0.0212 (6)	0.0319 (7)	−0.0030 (5)	0.0068 (5)	0.0025 (5)
C6	0.0231 (6)	0.0234 (6)	0.0260 (6)	0.0020 (5)	0.0040 (5)	0.0037 (5)
C10	0.0294 (7)	0.0242 (6)	0.0260 (6)	0.0006 (6)	0.0054 (6)	0.0076 (5)

### (2) Geometric parameters (Å, º)

**Table:**

O1—C7	1.3785 (16)	C5—H5	0.92 (3)
O1—C2	1.3963 (16)	C4—H4	1.01 (2)
C7—C8	1.3953 (18)	C12—C11	1.3946 (18)
C7—C12	1.3915 (18)	C12—H12	0.92 (2)
C2—C3	1.3870 (17)	C11—C10	1.392 (2)
C2—C1	1.386 (2)	C11—H11	0.94 (3)
C8—C9	1.388 (2)	C1—C6	1.392 (2)
C8—H8	0.99 (2)	C1—H1	0.93 (2)
C3—C4	1.3929 (19)	C9—C10	1.395 (2)
C3—H3	0.99 (2)	C9—H9	0.96 (3)
C5—C4	1.391 (2)	C6—H6	0.99 (3)
C5—C6	1.393 (2)	C10—H10	0.96 (2)
			
C7—O1—C2	117.93 (10)	C7—C12—C11	118.96 (12)
O1—C7—C8	115.26 (11)	C7—C12—H12	121.6 (12)
O1—C7—C12	123.79 (11)	C11—C12—H12	119.4 (12)
C12—C7—C8	120.95 (12)	C12—C11—H11	119.9 (15)
C3—C2—O1	119.23 (12)	C10—C11—C12	120.80 (13)
C1—C2—O1	118.80 (12)	C10—C11—H11	119.3 (14)
C1—C2—C3	121.88 (12)	C2—C1—C6	118.87 (12)
C7—C8—H8	119.5 (13)	C2—C1—H1	120.4 (14)
C9—C8—C7	119.30 (13)	C6—C1—H1	120.7 (13)
C9—C8—H8	121.2 (13)	C8—C9—C10	120.64 (13)
C2—C3—C4	118.65 (12)	C8—C9—H9	118.4 (15)
C2—C3—H3	118.9 (14)	C10—C9—H9	120.8 (15)
C4—C3—H3	122.5 (14)	C5—C6—H6	120.2 (15)
C4—C5—C6	119.84 (13)	C1—C6—C5	120.27 (13)
C4—C5—H5	120.1 (15)	C1—C6—H6	119.5 (15)
C6—C5—H5	120.0 (15)	C11—C10—C9	119.35 (13)
C3—C4—H4	119.9 (13)	C11—C10—H10	120.5 (15)
C5—C4—C3	120.50 (12)	C9—C10—H10	120.2 (15)
C5—C4—H4	119.5 (14)		
			
O1—C7—C8—C9	−178.71 (13)	C2—C3—C4—C5	0.0 (2)
O1—C7—C12—C11	178.53 (12)	C2—C1—C6—C5	0.0 (2)
O1—C2—C3—C4	−176.58 (12)	C8—C7—C12—C11	−0.92 (19)
O1—C2—C1—C6	176.58 (12)	C8—C9—C10—C11	−0.4 (2)
C7—O1—C2—C3	−90.12 (15)	C3—C2—C1—C6	0.0 (2)
C7—O1—C2—C1	93.20 (15)	C4—C5—C6—C1	−0.1 (2)
C7—C8—C9—C10	−0.1 (2)	C12—C7—C8—C9	0.79 (19)
C7—C12—C11—C10	0.4 (2)	C12—C11—C10—C9	0.3 (2)
C2—O1—C7—C8	177.95 (12)	C1—C2—C3—C4	0.0 (2)
C2—O1—C7—C12	−1.53 (18)	C6—C5—C4—C3	0.0 (2)
